# Azadirachtin Attenuates Lipopolysaccharide-Induced ROS Production, DNA Damage, and Apoptosis by Regulating JNK/Akt and AMPK/mTOR-Dependent Pathways in Rin-5F Pancreatic Beta Cells

**DOI:** 10.3390/biomedicines9121943

**Published:** 2021-12-18

**Authors:** Annie John, Haider Raza

**Affiliations:** Department of Biochemistry and Molecular Biology, College of Medicine and Health Sciences, United Arab Emirates University, Al Ain P.O. Box 17666, United Arab Emirates; anniej@uaeu.ac.ae

**Keywords:** pancreatic cells, LPS, azadirachtin, ROS, apoptosis, autophagy

## Abstract

Pancreatic inflammation and the resulting cellular responses have been implicated in pancreatitis, diabetes, and pancreatic cancer. Inflammatory responses due to the bacterial endotoxin, lipopolysaccharide (LPS), have been demonstrated to alter cellular metabolism, autophagy, apoptosis, and cell proliferation in different cell populations, and hence increases the risks for organ toxicity including cancer. The exact molecular mechanism is however not clear. In the present study, we investigated the role and mechanism of an antioxidant, azadirachtin (AZD), a limonoid extracted from the neem tree (*Azadirachta indica*), against LPS-induced oxidative stress in the pancreatic β-cell line, Rin-5F. We demonstrated that cells treated with LPS (1 µg/mL for 24 h) showed increased reactive oxygen species (ROS) production, DNA damage, cell cycle arrest, and apoptosis. Our results also showed that LPS induced alterations in the AMP-activated protein kinase (AMPK)/mammalian target of rapamycin (mTOR) pathways, suppressing autophagy and augmenting apoptosis. Treatment with Azadirachtin (25 µM for 24 h), on the other hand, rendered some degree of protection to the pancreatic cells from apoptosis by inducing the autophagy signals required for cell survival. These results may have significance in elucidating the mechanisms of pancreatic β-cell survival and death by balancing the molecular communication between autophagy and apoptosis under inflammatory and pathological conditions.

## 1. Introduction

Lipopolysaccharide (LPS), a bacterial endotoxin, induces septic shock, which may result in multiple organ dysfunction. The release of inflammatory mediators, such as tumor necrosis factor (TNF-α) and interleukins are considered to be the main triggers inducing cellular response and organ dysfunction [1]. These pro-inflammatory mediators are also responsible for initiating the cascade of secondary factors and signaling of autophagy, apoptosis, or cellular proliferation [2,3,4,5,6]. Homeostasis of pro-inflammatory and anti-inflammatory responses have also been implicated in a number of diseases [7,8]. Inflammatory stress promotes oxidative stress and vice versa. A high-fat diet can cause alterations in gut microbiota and induce up to three-fold lipopolysaccharide production, contributing to increased oxidative and ER stress, glucotoxicity, and lipotoxicity-associated complications, resulting in decreased pancreatic functions and triggering type 2 diabetes (T2D) development [9,10,11]. LPS-induced pancreatitis and β-cell mass destruction have been reported to be due to increased reactive nitrogen species and inflammatory signaling [12,13,14].

Using both in vivo animal models for type1 and type 2 diabetes [15,16,17,18,19] as well as in vitro models for glucolipotoxicity in different cellular models [20,21,22,23], our laboratory has demonstrated the significance of inflammation, oxidative stress, redox homeostasis, mitochondrial dysfunction, and reprogramming of energy metabolism in diabetes. The aim of the present study was to identify protective mechanisms of a potent anti-inflammatory compound, isolated from neem, on LPS-induced inflammation in a cell culture system using the insulin-secreting pancreatic beta cell, Rin-5F.

Azadirachtin (AZD), a phytochemical limonoid extracted from neem (*Azadirachta indica*) leaves and fruit, with known anti-inflammatory properties, has been reported to be beneficial in a number of inflammatory and oxidative stress-related abnormalities for more than 2000 years. In many degenerative disorders including diabetes, and various types of cancers including pancreatic cancer, the potential of neem plant extract is considered to be due to the abundant presence of the active limonoid, azadirachtin. Azadirachtin and related limonoids have been shown to protect β-cells from oxidative and inflammatory stress-induced cytotoxicity by manipulating the cell signalling kinases and anti-inflammatory responses [24,25,26,27,28]. The present study focuses on the identification of biochemical markers in Rin-5F cells affected by LPS treatment and the mechanism of protection of cytotoxicity by AZD. We report here that AZD alters β-cells’ responses towards oxidative/inflammatory stress by regulating cellular DNA damage, cell proliferation, and cellular signalling pathways regulating autophagy and apoptosis.

## 2. Materials and Methods

### 2.1. Materials

Azadirachtin (AZD, # A7430)), LPS (# L6511), 3-(4,5-dimethylthiazol-2-yl)-2,5-diphenyltetrazolium bromide (MTT, # M2128), propidium iodide (# P4170), Hoechst 33,342 (# B2261), and cellular DNA fragmentation ELISA kits (# 11 585 045 001) were purchased from Sigma-Aldrich (St Louis, MO, USA). 2,7-Dichlorofluorescein diacetate (DCFDA, # D399) was purchased from Molecular Probes, Inc. (Eugene, OR, USA). Kits for caspase-3 (# BF3100) and -8 assays (# K113-25) were procured from R&D Systems (Minneapolis, MN, USA). Apoptosis detection kits (# 556547) for flow cytometry were purchased from BD Pharmingen (BD Biosciences, San Jose, CA, USA). Rin-5F cells (#CRL-2058) were obtained from the American Type Culture Collection (Manassas, VA, USA). Polyclonal antibodies against cleaved caspase-3 (# 9661), poly (ADP-ribose) polymerase (PARP, # 9542)), autophagy-related protein (Atg5, # 8540)), microtubule-associated light chain 3 (LC3, # 2775), SQSTM1/p62 (# 5114), c-Jun N-terminal kinase (SAPK/JNK, # 9252), phosphorylated c-Jun N-terminal kinase (p-SAPK/JNK, # 9255), protein kinase B (AKT, # 4691), phosphorylated protein kinase B (p-AKT, #4060), AMP-activated protein kinaseα (AMPKα, #2532), phosphorylated AMP-activated protein kinaseα (p-AMPKα, # 2531), mammalian target of rapamycin (mTOR, # 2972), and phosphorylated mammalian target of rapamycin (p-mTOR, # 2971) were purchased from Cell Signaling Technology, Inc. (Danvers, MA, USA). Monoclonal antibodies against heat shock protein (Hsp-70, #H 5147) were purchased from Sigma-Aldrich (St Louis, MO, USA) while heme oxygenase-1 (HO-1, #ab 13243) was from Abcam (Cambridge, England, UK) and cyclin B1 (#sc-7393), p21 (#sc-6246), and β-actin (#sc-47778) from Santa Cruz Biotechnology Inc. (Santa Cruz, CA, USA).

### 2.2. Methods

#### 2.2.1. Cell Culture and Treatment

Rin-5F cell line is an insulin-secreting, epithelial pancreatic cell line derived from rat islet beta cells. The cells were cultured in RPMI-1640 GlutaMax medium supplemented with 10% foetal bovine serum in the presence of 5% CO_2_—95% air at 37 °C as described before [20,21,29]. Cells cultured to 80% confluence were treated with 1 µg/mL of the bacterial endotoxin, LPS for 24 h, based on literature reports and our previous studies [22,30,31]. In some cases, cells were treated with 25 µM azadirachtin for 24 h alone or in combination with LPS. After the desired time of treatment, cells were harvested, washed with PBS (pH 7.4), and homogenized and lysed in the appropriate lysis buffers required for the assays as described before [20,21,29].

#### 2.2.2. Cell Viability, DNA Damage and Apoptosis Assays

MTT assay was used to determine the mitochondrial dehydrogenase-based cellular viability. Briefly, cells treated with LPS and/or AZD were assessed for cell viability by the reduction of MTT dye to form insoluble purple formazan crystals, which were dissolved in acidified alcohol and the viable cells quantitated using an ELISA reader (TECAN Infinite M200 PRO, Austria) at 550 nm as described before [20,21].

DNA damage by apoptosis was assessed using Hoechst 33,342 dye staining of fragmented nuclei. Briefly, cells grown on coverslips were treated with LPS and/or AZD for 24 h and fixed with 3.7% formaldehyde. The cells were then stained with Hoechst 33,342 and observed by fluorescence microscopy (Olympus Corporation, Tokyo, Japan). Apoptotic cells showed fragmented nuclei. Oxidative stress–induced DNA damage was also measured after electrophoresis of DNA from LPS and/or AZD–treated cells and staining with ethidium bromide. 

Cellular DNA damage was also measured using the cellular DNA fragmentation kit (Sigma-Aldrich, St Louis, MO, USA) as per the vendor’s protocol. Briefly, cells were grown to confluency and cell count adjusted to around 2 × 10^5^ cells/mL medium and labeled with 5′-bromo-2′-deoxy-uridine (BrdU, used as a metabolic labeling agent by the nuclear DNA of target cells). After an overnight incubation at 37 °C, cells were centrifuged and resuspended in BrdU-free culture medium. The BrdU-labeled cells (1 × 10^5^ cells/mL) were then treated with LPS and/or AZD for 24 h. After treatment, the cells were lysed to extract the apoptotic DNA fragments from the cytoplasm. The cell extracts were then transferred to 96-well microtiter plates, which were pre-coated with anti-DNA antibody overnight and treated with blocking buffer to block all the non-specific binding sites. The plates with the cell extracts were incubated for 90 min at room temperature followed by washing the wells. The DNA in the samples were then fixed and denatured and then treated with anti-BrdU-peroxidase conjugate solution. After an incubation for 90 min at room temperature, a substrate was added and incubated in the dark until color development was sufficient, stop solution was added, and measurement was taken at 450 nm using a plate reader (TECAN Infinite M200 PRO, Austria).

Apoptosis in the LPS and/or AZD–treated cells were measured using a Becton Dickinson FACSCanto II analyser (BD Pharmingen, BD Biosciences, San Jose, CA, USA) as described before [22,31]. Briefly, Rin-5F cells were treated with LPS and/or AZD for 24 h and re-suspended in binding buffer (10 mM HEPES, pH 7.4, 140 mM NaCl, 2.5 mM CaCl_2_) at a concentration of 1 × 10^6^ cells/mL. A fraction of this cell suspension (1 × 10^5^ cells) was then incubated with FITC-conjugated Annexin V and propidium iodide. After 15 min, binding buffer was added and viable, apoptotic and necrotic cells were measured by flow cytometry. 

Caspase-3 and caspase-8 activities were measured in the cell lysate using the caspase-specific peptide substrates, Ac-Asp-Glu-Val-Asp (DEVD) and Ac-Ile-Glu-Thr-Asp (IETD) respectively, conjugated to the chromophore, p-nitroanaline. Cleavage of this peptide by the respective caspases releases the chromophore, which is measured colorimetrically at a wavelength of 405 nm as recommended in the vendor’s protocol (R&D Systems) as described before [20,29].

#### 2.2.3. Measurement of Intracellular Reactive Oxygen Species (ROS)

DCFDA, a cell permeable probe, which measures peroxides preferentially was used to measure the intracellular ROS production. LPS and/or AZD-treated cells were incubated with DCFDA and the fluorescence analyzed fluorometrically using the ELISA reader (TECAN Infinite M 200 PRO, Austria), microscopically using the Olympus fluorescent microscope (Olympus Corporation, Tokyo, Japan) and by flow cytometry using the FACSCanto II Flow Cytometer (Beckton Dickinson, San Jose, CA, USA) as described before [21,32]. 

#### 2.2.4. Cell Cycle Analysis by PI Staining

For cell cycle analysis, Rin-5F cells were fixed with 70% ethanol, treated with renatured RNaseA and stained with propidium iodide (PI) after treatment with LPS and/or AZD for 24 h and the different stages of cell cycle analyzed by flow cytometry as described by Piazza et al. [33]. The total DNA content was quantified at an excitation wavelength of 488 nm and detection wavelength of 620 nm using the FACSCanto II Flow Cytometer (Beckton Dickinson, San Jose, CA, USA) and the data analyzed using ModFit LT 3.2 software (Verity Software House; Topsham, ME, USA). Results are shown as representative histograms (from three repetitive experiments) showing percentage DNA distribution in each phase of the cell cycle.

#### 2.2.5. SDS-PAGE and Western Blot Analysis

Total cell extracts (25–50 µg) from control and LPS and/or AZD-treated Rin-5F cells were separated by 12% SDS-PAGE [34] and electrophoretically transferred onto nitrocellulose membrane by Western blotting [35]. Transferred proteins were checked by reversible Ponceau S staining for equal loading and then probed with primary antibodies against cleaved caspase-3, PARP, Hsp-70, HO-1, Atg-5, LC3, p62, Akt, p-Akt, mTOR, p-mTOR, and JNK and p-JNK. Immunoreactive bands were visualized using the appropriate conjugated secondary antibodies. Equal loading of protein was confirmed using beta-actin as loading control. Proteins were visualized by enhanced chemiluminescence using the Sapphire Biomolecular Imager (Azure biosystems, Dublin, CA, USA) or using X-ray films. Relative band intensity was quantified using Image Studio Lite Ver.5.2 (LI-COR Biosciences, Lincoln, NE, USA) and expressed as relative ratios normalized to beta-actin or its respective total proteins as appropriate.

#### 2.2.6. Statistical Analysis

Values shown are expressed as mean ± SD of at least three individual repetitive experiments. Statistical significance of the data was assessed using SPSS software (version 23) by analysis of variance (ANOVA) followed by least significant difference (LSD) post-hoc analysis for comparison between the different groups. *p*-value < 0.05 obtained after post-hoc analysis was considered statistically significant.

## 3. Results

### 3.1. Effects of LPS and AZD on Cell Viability, Apoptosis and Cell-Cycle Arrest

**Figure 1:**
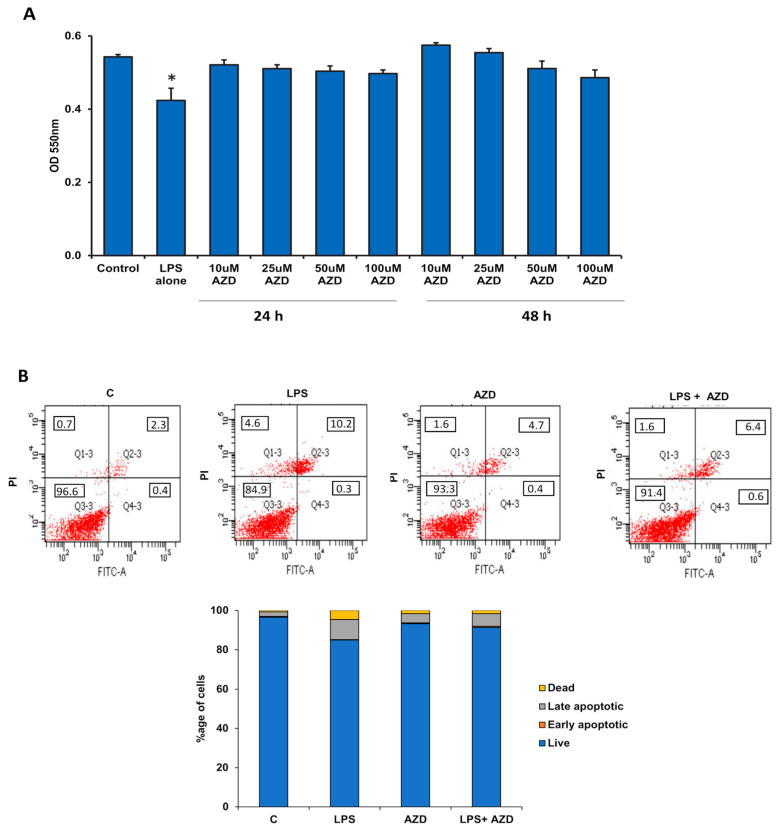

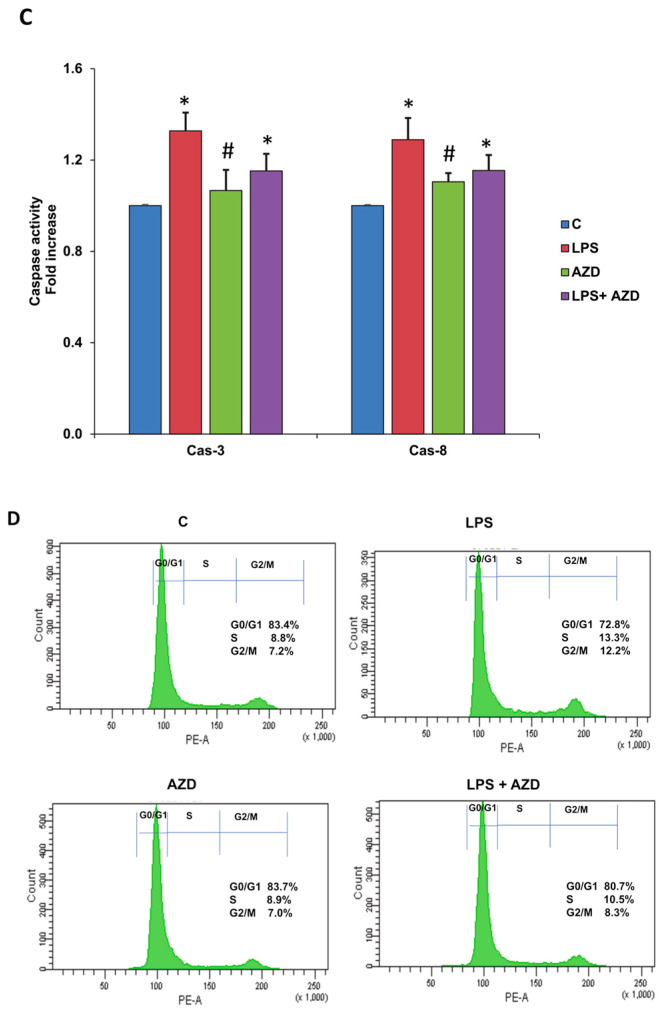
LPS-induced effects on cell viability, apoptosis, and cell-cycle regulation in Rin-5F cells. Rin-5F cells were treated with LPS alone (1 µg/mL) for 24 h and with different doses of AZD (10 µM to 100 µM) for different time intervals (24 h and 48 h), and MTT assay performed to assess cell viability (**A**). Apoptosis was measured in the cells treated with LPS (1 µg/mL) with or without AZD (25 µM) for 24 h by flow cytometry (**B**). A representative dot plot showing percentage of cells in the individual quadrants from three individual repetitive experiments is shown, and the average values are represented as a stacked column chart. Activities of caspases-3 and -8 were measured in treated and untreated cells colorimetrically using their respective substrates and the chromophore released was measured colorimetrically at a wavelength of 405 nm (**C**). Results are expressed as mean ± SD of three individual repetitive experiments. Asterisks indicate significant differences fixed at *p* ≤ 0.05 (* indicates significant difference relative to control untreated cells, whereas # indicates significant difference relative to LPS-treated cells). Cell cycle analysis was performed by staining the cells with propidium iodide, and the fluorescence was quantitated using the FACSCanto II Flow Cytometer at an excitation wavelength of 48 m with detection at 620 nm. Cell cycle data from the resulting histograms were analyzed with ModFit LT 3.2 software (Verity Software House; Topsham, ME) and expressed as percentage DNA distribution in each phase from three individual repetitive experiments. A representative histogram with DNA distribution in each phase is shown in (**D**).

To check the toxicity and effects on cell viability, Rin-5F cells were treated with LPS alone (1 µg/mL) for 24 h and with different doses of AZD (10 µM to 100 µM) for different time intervals (24 h and 4) and cell viability assessed by MTT assay. Rin-5F cells showed almost 22% reduction in cell viability (Figure 1A) after treatment with LPS alone (1 µg/mL) for 24 h. On the other hand, different doses of AZD (10 µM to 100 µM) caused no significant alterations in cell viability after 24 h or 48 h of treatments. These results suggest that AZD is not toxic to the pancreatic β-cells under the present culture system conditions and hence we used 25 µM of AZD for 24 h in the follow-up experiments.

Next, we investigated the effects of LPS and/or AZD on apoptosis of Rin-5F cells. As shown in Figure 1B, cells treated with 1 µg/mL LPS exhibited around 15% late apoptotic or necrotic cell death, whereas treatment with 25 µM AZD for 24 h alone or in combination with LPS resulted in less than 10% late apoptosis. These results suggest some degree of protection by AZD on LPS-induced cell death.

In support, caspase-3 and caspase-8 activities were also found to be significantly increased (about 30%) by LPS alone (Figure 1C), while AZD alone or in combination with LPS caused only a slight increase in the caspase activities. Caspase activation by AZD alone was significantly lower than that with LPS alone, showing the non-toxic effect of AZD at this concentration. AZD also helped in moderately reducing the activation of caspases, again suggesting a protective effect of AZD.

To further check whether LPS induces cell cycle arrest, we performed cell cycle analysis by staining the cells with propidium iodide and the fluorescence was quantitated using the FACSCanto II Flow Cytometer (Beckton Dickinson, San Jose, CA, USA). As shown in Figure 1D, LPS-treated cells showed an accumulation of cells in the S and G2/M phase (G0/G1-72.8%, S-13.3%, and G2/M-12.2%) compared with the control untreated cells (G0/G1-83.4%, S-8.8%, and G2/M-7.2%). AZD alone showed (G0/G1-83.7%, S-8.9%, and G2/M-7.0%) whereas in combination with LPS, the cell cycle distribution was G0/G1-80.7%, S-10.5%, and G2/M-8.3%. This again suggests the apoptotic effect of LPS and confirms the protective effect of AZD.

### 3.2. LPS-Induced ROS Generation and Attenuation by AZD

**Figure 2:**
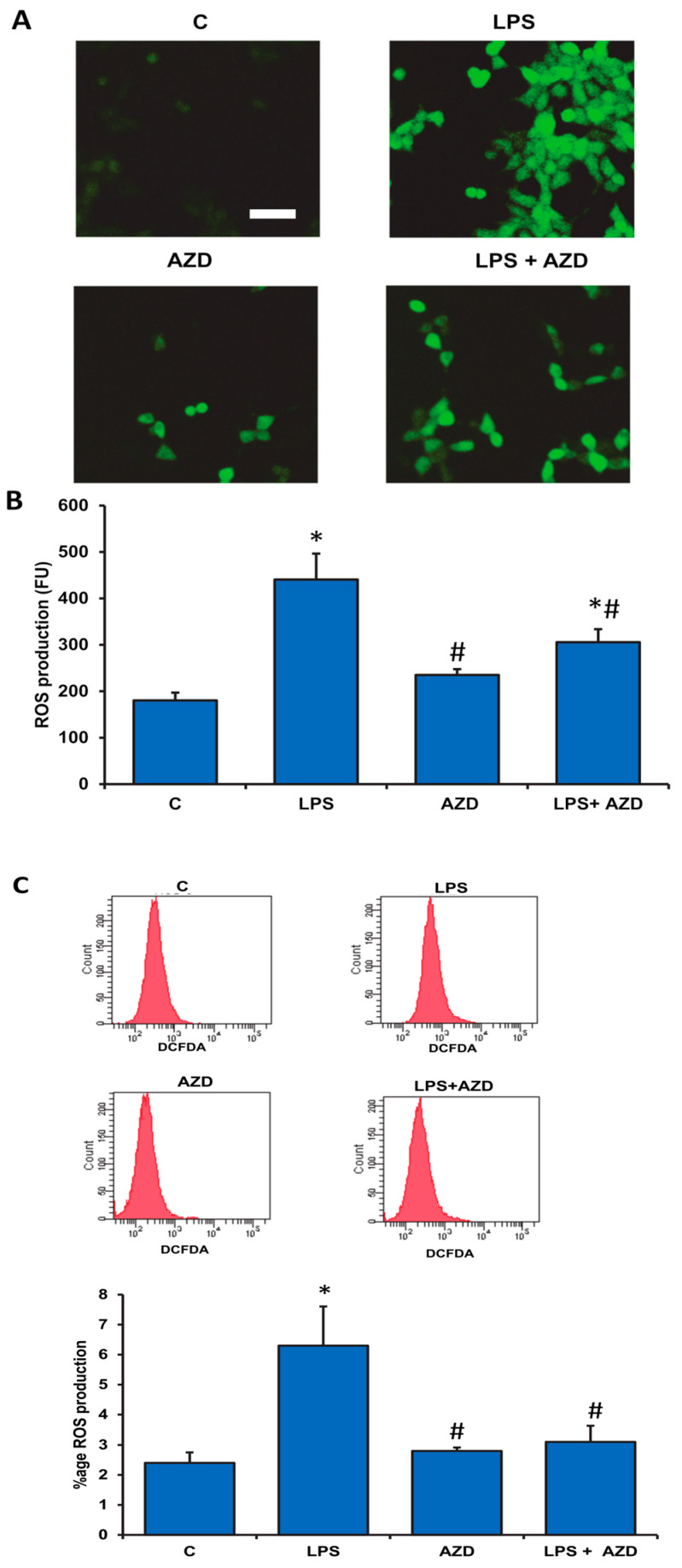
LPS-induced ROS generation in Rin-5F cells. ROS generation in Rin-5F cells treated with LPS alone (1 µg/mL) with or without AZD (25 µM) for 24 h was demonstrated using DCFDA probe microscopically (**A**) on cells grown on cover slips, fluorimetrically (**B**) using the ELISA reader, and by flow cytometry (**C**) using the BD FACSCanto II Flow Cytometer at an excitation wavelength of 488 nm and an emission wavelength of 525 nm, which showed increased ROS production after LPS treatment. Scale bar in (2A) indicates 50 µm. Results are expressed as mean ± SD of three individual repetitive experiments. Asterisks indicate significant differences fixed at *p* ≤ 0.05 (* indicates significant difference relative to control untreated cells, whereas # indicates significant difference relative to LPS-treated cells).

ROS generation has been known to play a major role in apoptotic cell death. DCFDA staining was performed in cells treated with LPS and/or AZD and analyzed by microscopy, fluorimetry, and flow cytometry. The microscopic analysis in Figure 2A shows increased ROS positive cells after LPS treatment in Rin-5F cells compared with AZD alone or in combination. A marked increase in ROS production after LPS treatment was also confirmed fluorometrically (Figure 2B). When LPS treated cells were incubated with AZD, a significant reduction in the production of DCFDA-sensitive ROS was observed.

FACS analysis also demonstrated a marked (three-fold) increase in ROS production after LPS treatment, while AZD treatment alone showed no appreciable production of ROS. LPS-induced ROS was significantly reduced in the presence of AZD (Figure 2C).

### 3.3. LPS-Induced DNA Damage

**Figure 3:**
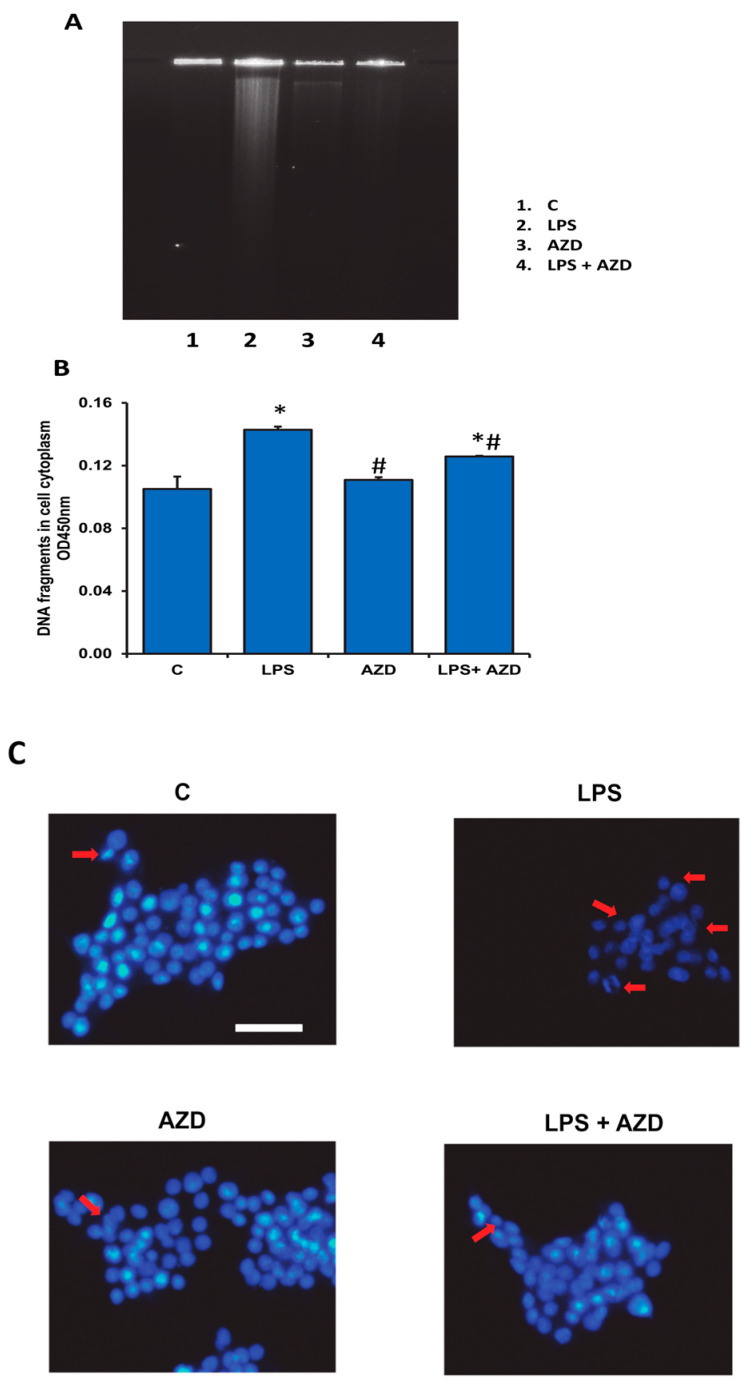
LPS-induced DNA damage in Rin-5F cells. DNA damage was assessed in Rin-5F cells treated with LPS alone (1 µg/mL) with or without AZD (25 µM) for 24 h. DNA break-down was confirmed using 2% agarose gel electrophoresis and staining with ethidium bromide (**A**). Cellular DNA damage was also assessed by ELISA using the cellular DNA fragmentation kit (**B**). Results are expressed as mean ± SD of three individual repetitive experiments. Asterisks indicate significant differences fixed at *p* ≤ 0.05 (* indicates significant difference relative to control untreated cells, whereas # indicates significant difference relative to LPS-treated cells). Staining of the nuclei was also performed using the Hoechst 33,342 dye and the slides observed under a fluorescence microscope (**C**). Scale bar indicates 50 µm. Red arrows indicate apoptotic cells showing damaged nuclei. Representative results from control and LPS alone and AZD with or without LPS from three individual repetitive experiments are shown.

DNA fragmentation has been known to be the ‘hallmark’ for apoptosis. So, to further confirm the apoptotic effect of LPS, we performed DNA fragmentation assay after treatment of Rin-5F cells with LPS and/or AZD. Agarose gel electrophoresis showed DNA breakdown after LPS treatment (Figure 3A). However, a significant reduction was observed when cells were treated with AZD in combination with LPS, suggesting a protection of DNA from LPS-induced cytotoxicity. Cellular DNA damage by ELISA using the cellular DNA fragmentation kit (Sigma, St Louis, MO, USA) also showed increased DNA fragments in the cellular cytoplasm in LPS-treated cells and significant reduction with AZD alone or in combination (Figure 3B). Similarly, Hoechst 33,342 DNA-staining of Rin-5F cells and microscopic visualization exhibited markedly reduced Hoechst-positive cells after LPS treatment in comparison with AZD alone or in combination (Figure 3C).

### 3.4. LPS-Induced Expression of Oxidative Stress and Apoptosis Markers

**Figure 4:**
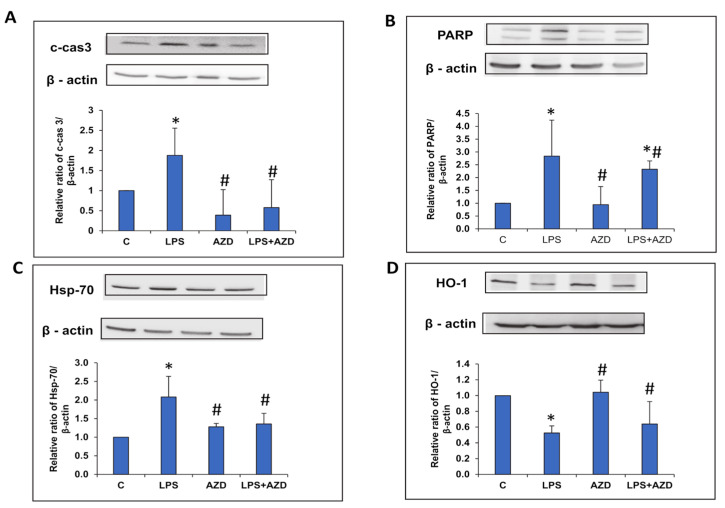
LPS-induced expression of oxidative stress markers. Protein extracts from Rin-5F cells treated with LPS with/without AZD were separated on 12% SDS-PAGE and transferred on to nitrocellulose paper by Western blotting and protein bands detected using the appropriate primary antibodies. Specific antibodies against cleaved caspase 3 (**A**), PARP (**B**), Hsp-70 (**C**), and HO-1 (**D**) were used to detect the respective proteins and visualized by enhanced chemiluminescence using the Sapphire Biomolecular Imager (Azure biosystems, Dublin U.S.A) or using X-ray films. Beta actin was used as a loading control. Histograms represent the relative ratios of the quantitated proteins normalized against the loading control. The figures are a representative of at least three individual repetitive experiments. Asterisks indicate significant differences fixed at *p* ≤ 0.05 (* indicates significant difference relative to control untreated cells, whereas # indicates significant difference relative to LPS-treated cells).

To further prove the oxidative stress and apoptosis caused by LPS, we checked the expression of oxidative stress, apoptosis, and autophagy markers. As shown in Figure 4A,B, LPS treatment markedly enhanced the expression of cleaved caspase 3 and PARP. AZD treatment in combination with LPS significantly reduced the expression of these apoptotic marker proteins. The expression of Hsp-70, an oxidative stress marker protein, was also increased after LPS treatment, which was normalized after AZD treatment close to control values (Figure 4C). On the other hand, the expression of HO-1, another redox marker protein, was significantly (almost 50%) reduced after LPS treatment, which came close to control levels after AZD treatment (Figure 4D). AZD treatment alone showed almost no change in the expression of these proteins when compared with control cells.

### 3.5. LPS-Induced Alterations in the Expression of Autophagy Markers

**Figure 5:**
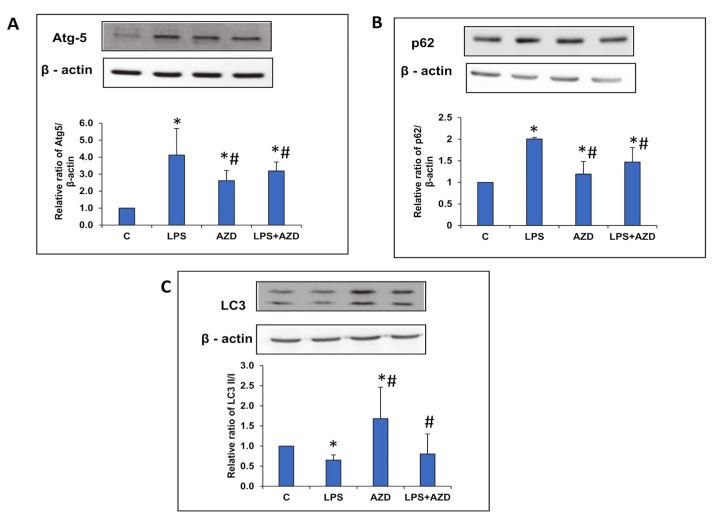
LPS-induced alterations in the expression of autophagy markers. Protein extracts from Rin-5F cells treated with LPS with/without AZD were separated on 12% SDS-PAGE and transferred on to nitrocellulose paper by Western blotting. Transferred proteins were incubated with primary antibodies against Atg-5 (**A**), p62 (**B**), and LC3 (**C**), and specific proteins visualized by enhanced chemiluminescence using the Sapphire Biomolecular Imager (Azure biosystems, Dublin USA) or using X-ray films. Beta actin was used as a loading control. Histograms represent the relative ratios of the quantitated proteins normalized against the loading control. The figures are representative of at least three individual repetitive experiments. Asterisks indicate significant differences fixed at *p* ≤ 0.05 (* indicates significant difference relative to control untreated cells, whereas # indicates significant difference relative to LPS-treated cells).

We further tried to investigate the regulation of autophagy by LPS. Figure 5A,B shows a four-fold increase in the expression Atg-5 protein and a two-fold increase in the expression of p62 protein respectively, after LPS treatment. AZD, on the other hand, significantly reduced the enhanced expression of these marker proteins. However, the expression of LC3 protein involved in the elongation of autophagosomes was decreased after LPS treatment, while AZD treatment alone enhanced the expression of this protein (Figure 5C). Treatment of cells with AZD in the presence of LPS showed moderate alterations on the expression of the proteins when compared with control cells. These results suggest a restricted autophagic machinery in Rin-5F cells after LPS treatment, perhaps resulting in their increased apoptosis under these conditions.

### 3.6. LPS–Induced Alterations of Cell Cycle Progression Markers

**Figure 6:**
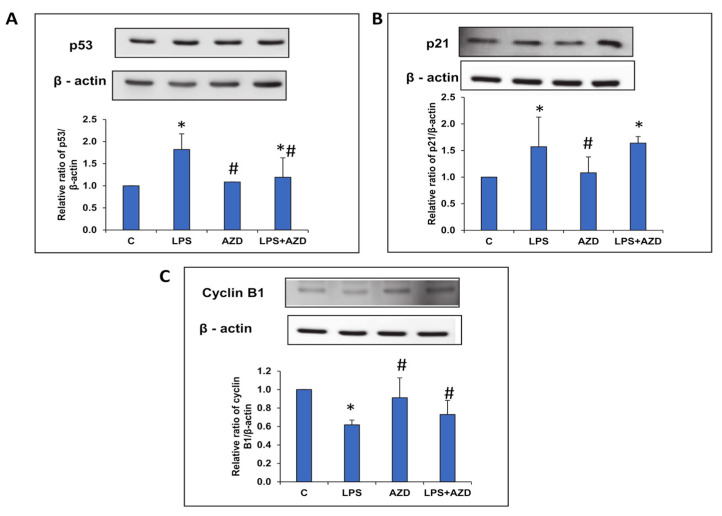
LPS-induced alterations in the expression of cell cycle regulatory markers. Protein extracts from Rin-5F cells treated with LPS with/without AZD were separated on 12% SDS-PAGE and transferred on to nitrocellulose paper by Western blotting. Transferred proteins were detected using specific antibodies against p53 (**A**), p21 (**B**), and cyclin B1 (**C**), and visualized by enhanced chemiluminescence using the Sapphire Biomolecular Imager (Azure biosystems, Dublin USA) or using X-ray films. Beta actin was used as loading control. Histograms represent the relative ratios of the quantitated proteins normalized against the loading control. The figures are representative of at least three individual repetitive experiments. Asterisks indicate significant differences fixed at *p* ≤ 0.05 (* indicates significant difference relative to control untreated cells, whereas # indicates significant difference relative to LPS-treated cells).

To confirm the cell cycle arrest observed by flow cytometry, we investigated the expression of p53 protein, known to suppress cell cycle progression and cause apoptosis and cell death. Our study demonstrated an increased expression of p53 (Figure 6A) after LPS treatment, which reduced significantly on treatment with AZD. We also observed an increase in the expression of its transcriptional target, p21, which can cause cell cycle arrest at the G1/S phase or G2/M phase (Figure 6B). We further studied the expression of cyclin B1 to confirm cell cycle arrest at the G2/M phase. Consistent with our cell cycle flow cytometry results, we observed an LPS-induced decrease in the expression of cyclin B1 protein, which was increased after AZD treatment, confirming cell cycle arrest at the G2/M phase (Figure 6C).

### 3.7. LPS-Induced Alteration of the JNK/Akt and AMPK/mTOR Signaling Pathways

**Figure 7:**
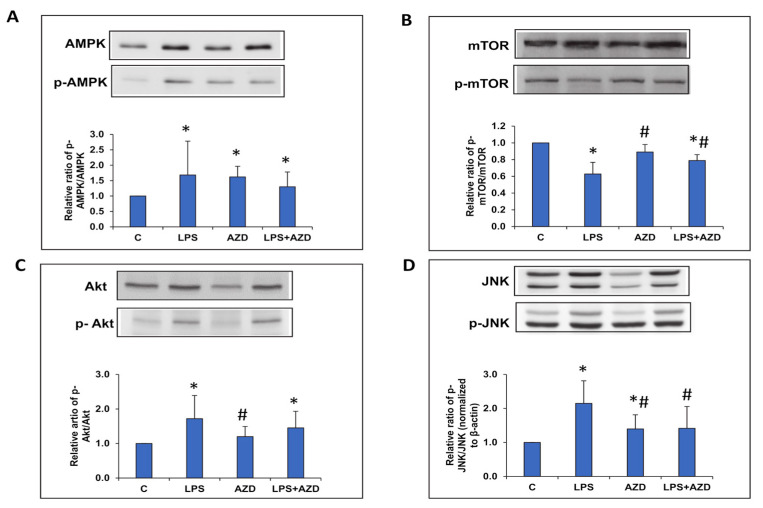
LPS-induced alterations in the expression of cell signaling proteins. Protein extracts from Rin-5F cells treated with LPS with/without AZD were separated on 12% SDS-PAGE and transferred on to nitrocellulose paper by Western blotting. Specific antibodies against AMPK/p-AMPK (**A**), mTOR/p-MTOR (**B**), Akt/p-Akt (**C**), and JNK/p-JNK (**D**) were used to detect the respective proteins. Beta actin was used as a loading control. Histograms represent the relative ratios of the phosphorylated proteins to total proteins normalized with the loading control. The figures are representative of at least three individual repetitive experiments. Asterisks indicate significant differences fixed at *p* ≤ 0.05 (* indicates significant difference relative to control untreated cells, whereas # indicates significant difference relative to LPS-treated cells).

We further investigated the correlation of apoptosis, ROS production, and autophagy to the expression of cell signaling markers after LPS and AZD treatment alone or in combination. As shown in Figure 7A, phosphorylation of the AMPK protein, a key energy sensor and regulator of autophagy/apoptosis, significantly increased after LPS treatment. This could be due to increased ATP consumption after LPS treatment, which could be the cause for the pathway leading towards apoptosis. Treatment with AZD, alone or in combination, also moderately increased the levels of p-AMPK, compared with the control cells. Concomitant to this, we observed a decrease in the phosphorylation of mammalian target of rapamycin (mTOR), which is also a sensor of metabolic signals (like reactive oxygen species, ATP level) after LPS treatment (Figure 7B). A significant increase in expression was observed after AZD treatment, though it was much lower compared with the control untreated cells. Treatment with AZD alone, however, showed no appreciable change. These results may suggest that cells are facing autophagic regulation and are going into catabolic mode to regulate energy metabolism after LPS treatment. Next, we checked the expression of phosphorylated Akt (p-Akt), which was significantly increased in Rin-5F cells treated with LPS (Figure 7C). No significant change was observed after AZD treatment, though the expression was significantly low in cells treated with AZD alone. A similar increase in the expression of ROS-sensitive JNK signaling protein was observed after LPS treatment, suggesting the activation of downward signaling pathways altering autophagy and apoptosis, which would determine the fate of cells under this type of oxidative/inflammatory stress condition (Figure 7D). Increased phosphorylation of JNK protein was also observed after AZD treatment, alone or in combination, though it was significantly lower compared with the LPS-induced cells.

## 4. Discussion

β-cells are metabolically highly active for the generation of ATP and insulin secretion by aerobic energy metabolism and mitochondrial respiratory function, especially in response to elevated blood glucose levels [36,37]. This renders β-cells susceptible to ROS production and oxidative stress. On the other hand, beta cells are also highly vulnerable towards oxidative stress due to insufficient antioxidant defenses such as lower expression of SOD, GSH-Px, and catalase in comparison with other tissues [38,39]. LPS induces oxidative stress and inflammatory stimuli, which may trigger the induction of autophagy and/or apoptosis. The final outcome of cell death or survival depends on the complex cross-talk between numerous apoptosis-related and autophagy-related proteins and signaling cascades.

Our previous studies, using Rin-5F cells, have shown that these cells exhibit increased inflammatory and oxidative stress responses under glucolipotoxicity conditions, as well as when treated with streptozotocin, a β-cell damaging diabetogenic agent [20,23,32]. In the present study, we further elucidated the molecular mechanism of increased oxidative/inflammatory stress after treating these cells with a bacterial endotoxin, LPS, alone or in combination with a non-toxic concentration of AZD, a known anti-inflammatory phytochemical limonoid extracted from neem. Our results demonstrated increased ROS production, caspase activation, DNA fragmentation, and cell cycle arrest, resulting in increased apoptosis in LPS-treated cells, which was attenuated after AZD treatment.

In order to elucidate the molecular mechanism of LPS-induced cell death and prevention by AZD, we further investigated the expression of proteins involved in oxidative stress, autophagy, apoptosis, and cell signaling cascades. These proteins positively or negatively regulate both autophagy and apoptosis. The net effect of whether cells are going to survive by induction of autophagy or go in to autophagy-induced cell death or apoptosis depends on the cell type, stimuli, and escape from cell death due to therapeutic/preventive treatments. Autophagy may play a synergistic role in cell death by providing a membrane-based intracellular platform for caspase processing, by inhibiting the apoptosis by removing apoptotic mitochondria for intrinsic apoptosis, or by degrading the caspases for extrinsic apoptotic pathways. Initially, autophagy functions as an adaptive response to stress, however, in the face of extreme or chronic stress, cells undergo autophagy cell death [40,41]. Our results show that LPS-induced cleavage of pro-caspase 3 and PARP to initiate apoptosis was markedly reduced after AZD treatment. This confirmed our observation that Rin-5F cells treated with LPS undergo oxidative stress, as seen by the increased production of ROS followed by DNA fragmentation. This was also supported by the increased expression of oxidative stress marker protein, Hsp-70. On the other hand, HO-1 expression, which is under the regulation of redox-regulating genes, was reduced in LPS-treated cells and enhanced after AZD treatment. HO-1 is a cytoprotective protein known to curtail cytotoxicity caused by oxidative stress and inflammatory reactions and by reducing ROS production [42].

Increased expression of apoptosis-regulating protein p53, was also observed after LPS treatment and AZD caused a reduction of LPS-induced p53. There have been studies suggesting the role of p53 both in extrinsic and intrinsic apoptosis. Increased expression of p53 triggers the expression of apoptotic proteins, resulting in increased apoptosis [43,44]. In the extrinsic pathway, cytoplasmic p53 activates caspase 8 and caspase 3. Our study also showed increased activities of caspase 8 and caspase 3 proteases after LPS treatment. There are reports showing that p53 activation can promote cell cycle arrest and apoptosis through transcription-independent mechanisms [45,46]. p53 is known to prevent cell cycle progression of cells with damaged DNA. Regarding DNA damage, p53 is activated, which in turn induces the production of p21 protein, which is a cyclin-dependent kinase (Cdk) inhibitor. Cdks are required for cell cycle progression from G1 to S phase and G2 to M phase. Thus, p53 could induce G1/S phase or G2/M phase cell cycle arrest depending on the cyclin inhibited [47]. This was confirmed in our present study, which revealed a greater proportion of cells in the S and G2/M phase after LPS treatment by flow cytometric analysis compared to the control cells, correlating with the inhibition of cyclin B1. The distribution normalized close to control levels after treatment with AZD. The increased activation of p53 after LPS treatment, accompanied by p21 accumulation and down-regulation of the cell cycle regulatory protein, cyclin B1, resulted in cell cycle arrest at the G2/M phase. Researchers have also shown that p53 represses autophagy through AMPK/mTOR pathways [48]. Our results showed activation in the expression of AMPK associated with a decrease in the expression of mTOR after LPS treatment. However, a decrease in autophagy and increase in apoptosis was observed in these cells. This is in agreement with a study that indicated that inactivation of mTOR can sensitize the proapoptotic response to bacterial infection [49].

We also showed an increased Akt activation/phosphorylation by LPS in Rin-5F cells, however, this was not inhibited after AZD treatment, suggesting the limited role of AZD in Akt-mediated regulation of autophagy/apoptosis/survival in Rin-5F cells under the present experimental conditions. AZD showed no effect on Akt expression in the control untreated cells as well. Also, our study showed an increased expression of AMPK and a decreased activation of mTOR after LPS treatment. There are a number of studies supporting our observation of LPS-induced alterations in AMPK/mTOR signaling various cellular systems [50,51,52,53]. We also observed a moderate increase in AMPK activation and a moderate decrease in mTOR phosphorylation after AZD treatment. AZD has also been shown to inhibits pancreatic cancer growth and metastasis through ROS-mediated apoptosis, and also induces autophagy and apoptosis via AKT/mTOR/Atg5 pathways [54,55]. mTOR signaling plays a major role in autophagy as well as in apoptosis, depending on the specific cellular conditions and downstream targets such as p53 [44]. These researchers have also shown that alterations in the Akt/mTOR pathways in cancer cells can also induce both autophagy and apoptosis.

Oxidative stress activates JNK1, thus promoting autophagy. However, constitutively active JNK1 prevents the initiation of autophagy. On the contrary, under extreme stress conditions, JNK1 induces apoptosis through caspase 3-dependent pathways. Thus, JNK cell signaling represents an important link between autophagy and apoptosis [56]. Our results showed an increased expression of p-JNK in LPS-treated cells, which was reduced after AZD treatment. AZD treatment alone also increased the expression of p-JNK, suggesting that AZD itself plays some role in inducing cell death or survival and that it may be related with the type of cell system and activation of other signaling cascades under stress conditions. Our results also demonstrate an altered expression of autophagy-regulating proteins, Atg-5, p62, and LC3. While Atg-5 and p62 expressions were increased after LPS treatment and inhibited after AZD treatment, the LC3 expression was repressed in Rin-5F cells after LPS treatment. AZD treatment alone, on the other hand, increased the expression of LC3 but not when treated along with LPS, suggesting some independent mechanism involved in the regulation of apoptosis or autophagy when treated alone or in combination with LPS. Further studies are needed to elucidate the exact mechanism of action of these proteins involved in the autophagy/apoptosis machinery. LC3 and p62 play important roles as receptors at the phagophore membrane to process protein aggregates and damaged mitochondria for engulfment. Under oxidative stress conditions and with increased ROS, inhibition of autophagy promotes p62, increases the level of caspases, and increases apoptosis [57]. Proteins regulating the elongation process of autophagy have also been shown to participate in the apoptotic pathway. Cleavage of Atg-5 contributes to apoptosis in various cell types. Moreover, N-terminal Atg-5 cleavage fragments promotes nuclear fragmentation and prevents LC3 accumulation, suggesting the ability of cleaved Atg-5 to induce apoptosis but not autophagy. Similarly, caspase-3-induced cleavage of autophagy-related proteins also induces apoptosis [58]. Autophagy has also been shown to be mutually associated and regulated by the cell cycle [59,60].

## 5. Conclusions

**Figure 8:**
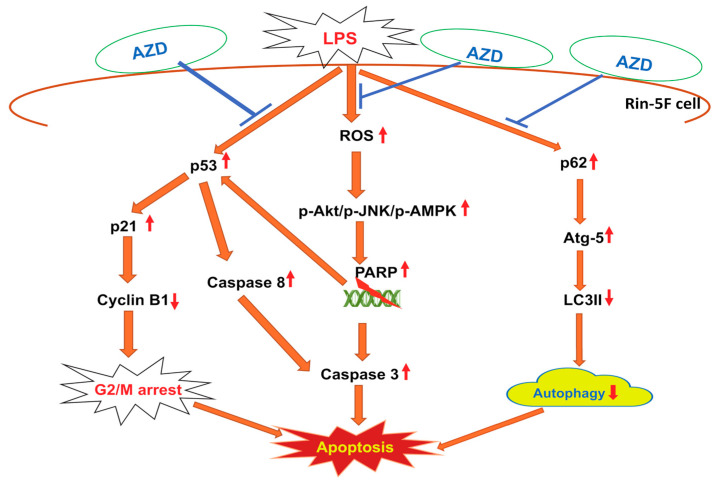
Schematic model representing the cross-talk between signaling proteins involved in the regulation of cell survival and death after LPS-induced oxidative stress and its attenuation by AZD in Rin-5F cells. LPS has been shown to cause increased oxidative stress; activation of the cell signaling kinases; DNA breakdown, causing p53 activation and leading to cell cycle arrest; and apoptosis by activation of caspases. LPS and AZD have also been shown to cause alterations in the expression of autophagy markers. As shown in the model, AZD gives some degree of protection by reducing oxidative stress and enhancing autophagy. (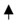 indicates activation whereas, 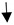 indicates a decrease, and 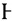 indicates inhibition).

Pancreatic β-cells have shown high sensitivity towards inflammatory and oxidative stresses. Cells exposed to bacterial endotoxin, LPS, responded by enhanced ROS, DNA degradation, cell cycle dysregulation, and cell death. When treated with an antioxidant phytochemical, AZD, these responses were diverted to repair the damages caused by inducing autophagy-type cellular defense. As shown in the schematic model (Figure 8), autophagy and apoptosis cross-regulate each other through an interconnecting network of cell-signaling proteins and autophagy- and apoptosis-related proteins. Our present study suggests a possible cross-talk between signaling proteins and may have therapeutic implications in better understanding the molecular mechanism of pancreatic β-cell survival and death under inflammatory and other pathological conditions, including cancer. Further studies on the recognition of the signal cascade and the resulting metabolic consequences will establish precisely the role of the molecular communication in determining the fate of cells.

## 6. Limitations

The main limitation is that this in-vitro study has been done on a single cell line from rats. Further studies on similar cell lines in humans as well as in vivo studies are required to confirm the therapeutic implications of this limonoid. Also, functional analysis needs to be performed to affirm the cross-talk between cell signaling proteins to understand better the molecular mechanism/pathway of pancreatic cell death and survival under pathological conditions.

## Data Availability

All data provided in the manuscript.
